# 5-Cyclo­hexyl-2-(3-fluoro­phen­yl)-3-methyl­sulfinyl-1-benzofuran

**DOI:** 10.1107/S1600536812008343

**Published:** 2012-03-03

**Authors:** Hong Dae Choi, Pil Ja Seo, Uk Lee

**Affiliations:** a Department of Chemistry, Dongeui University, San 24 Kaya-dong Busanjin-gu, Busan 614-714, Republic of Korea; b Department of Chemistry, Pukyong National University, 599-1 Daeyeon 3-dong, Nam-gu, Busan 608-737, Republic of Korea

## Abstract

In the title compound, C_21_H_21_FO_2_S, the cyclo­hexyl ring adopts a chair conformation. The 3-fluoro­phenyl ring makes a dihedral angle of 38.38 (6)° with the mean plane [r.m.s. deviation = 0.010 (1) Å] of the benzofuran fragment. In the crystal, mol­ecules are linked by weak C—H⋯O hydrogen bonds.

## Related literature

For background information and the crystal structures of related compounds, see: Choi *et al.* (2011*a* ▶,*b* ▶).
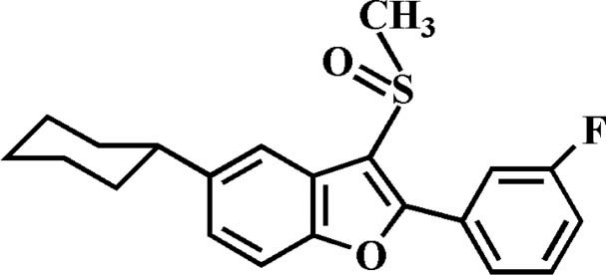


## Experimental

### 

#### Crystal data

C_21_H_21_FO_2_S
*M* *_r_* = 356.44
Orthorhombic, 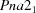

*a* = 12.7767 (10) Å
*b* = 13.0764 (10) Å
*c* = 10.7711 (8) Å
*V* = 1799.6 (2) Å^3^
*Z* = 4
Mo *K*α radiation
μ = 0.20 mm^−1^
*T* = 173 K
0.34 × 0.24 × 0.20 mm

#### Data collection

Bruker SMART APEXII CCD diffractometer
Absorption correction: multi-scan (*SADABS*; Bruker, 2009 ▶) *T*_min_ = 0.935, *T*_max_ = 0.961
17893 measured reflections
4503 independent reflections
4036 reflections with *I* > 2σ(*I*)
*R*_int_ = 0.058

#### Refinement

*R*[*F* ^2^ > 2σ(*F* ^2^)] = 0.041
*wR*(*F* ^2^) = 0.096
*S* = 1.03
4503 reflections
227 parameters
1 restraint
H-atom parameters constrained
Δρ_max_ = 0.33 e Å^−3^
Δρ_min_ = −0.25 e Å^−3^
Absolute structure: Flack (1983 ▶), 2131 Friedel pairs
Flack parameter: −0.07 (7)

### 

Data collection: *APEX2* (Bruker, 2009 ▶); cell refinement: *SAINT* (Bruker, 2009 ▶); data reduction: *SAINT*; program(s) used to solve structure: *SHELXS97* (Sheldrick, 2008 ▶); program(s) used to refine structure: *SHELXL97* (Sheldrick, 2008 ▶); molecular graphics: *ORTEP-3* (Farrugia, 1997 ▶) and *DIAMOND* (Brandenburg, 1998 ▶); software used to prepare material for publication: *SHELXL97*.

## Supplementary Material

Crystal structure: contains datablock(s) global, I. DOI: 10.1107/S1600536812008343/bh2416sup1.cif

Structure factors: contains datablock(s) I. DOI: 10.1107/S1600536812008343/bh2416Isup2.hkl

Supplementary material file. DOI: 10.1107/S1600536812008343/bh2416Isup3.cml

Additional supplementary materials:  crystallographic information; 3D view; checkCIF report

## Figures and Tables

**Table 1:** Hydrogen-bond geometry (Å, °)

*D*—H⋯*A*	*D*—H	H⋯*A*	*D*⋯*A*	*D*—H⋯*A*
C21—H21*B*⋯O2^i^	0.98	2.30	3.276 (3)	176

Symmetry code: (i) 
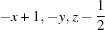
.

**Fig. 1.**
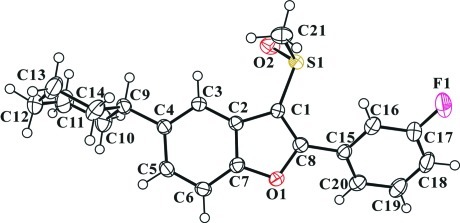
The molecular structure of the title compound with displacement ellipsoids drawn at the 50% probability level. H atoms are presented as small spheres of arbitrary radius.

## supplementary crystallographic information

### Comment

As a part of our ongoing study of 5-cyclohexyl-3-methylsulfinyl-1-benzofuran derivatives containing either 2-phenyl (Choi *et al.*, 2011*a*) or 2-(4-fluorophenyl) (Choi *et al.*, 2011*b*) substituents, we report herein the crystal structure of the title compound.

In the title molecule (Fig. 1), the benzofuran unit is essentially planar, with a mean deviation of 0.010 (1) Å from the least-squares plane defined by the nine constituent atoms. The cyclohexyl ring is in the chair form. The dihedral angle between the 3-fluorophenyl ring and the mean plane of the benzofurn fragment is 38.38 (6)°. The crystal packing is stabilized by weak intermolecular C–H···O hydrogen bonds (Table 1).

### Experimental

77% 3-Chloroperoxybenzoic acid (224 mg, 1.0 mmol) was added in small portions to a stirred solution of 5-cyclohexyl-2-(3-fluorophenyl)-3-methylsulfanyl-1-benzofuran (306 mg, 0.9 mmol) in dichloromethane (30 mL) at 273 K. After being stirred at room temperature for 4 h., the mixture was washed with saturated sodium bicarbonate solution and the organic layer was separated, dried over magnesium sulfate, filtered and concentrated at reduced pressure. The residue was purified by column chromatography (hexane-ethyl acetate, 1:1 *v*/*v*) to afford the title compound as a colorless solid [yield 76%, m.p. 443-444 K; *R*_f_ = 0.55 (hexane-ethyl acetate, 1:1 *v*/*v*)]. Single crystals suitable for X-ray diffraction were prepared by slow evaporation of a solution of the title compound in acetone at room temperature.

### Refinement

All H atoms were positioned geometrically and refined using a riding model, with C–H = 0.95 Å for aryl, 1.0 Å for methine, 0.99 Å for methylene and 0.98 Å for methyl H atoms, respectively. *U*_iso_(H) =1.2*U*_eq_(C) for aryl, methine, and methylene, and 1.5*U*_eq_(C) for methyl H atoms.

### Crystal data

**Table:**

C_21_H_21_FO_2_S	*D*_x_ = 1.316 Mg m^−^^3^
*M**_r_* = 356.44	Melting point: 443 K
Orthorhombic, *P**n**a*2_1_	Mo *K*α radiation, λ = 0.71073 Å
Hall symbol: P 2c -2n	Cell parameters from 4473 reflections
*a* = 12.7767 (10) Å	θ = 2.5–28.1°
*b* = 13.0764 (10) Å	µ = 0.20 mm^−^^1^
*c* = 10.7711 (8) Å	*T* = 173 K
*V* = 1799.6 (2) Å^3^	Block, colourless
*Z* = 4	0.34 × 0.24 × 0.20 mm
*F*(000) = 752	

### Data collection

**Table:**

Bruker SMART APEXII CCD diffractometer	4503 independent reflections
Radiation source: rotating anode	4036 reflections with *I* > 2σ(*I*)
Graphite multilayer monochromator	*R*_int_ = 0.058
Detector resolution: 10.0 pixels mm^-1^	θ_max_ = 28.4°, θ_min_ = 2.2°
φ and ω scans	*h* = −17→17
Absorption correction: multi-scan (*SADABS*; Bruker, 2009)	*k* = −17→16
*T*_min_ = 0.935, *T*_max_ = 0.961	*l* = −14→14
17893 measured reflections	

### Refinement

**Table:**

Refinement on *F*^2^	Secondary atom site location: difference Fourier map
Least-squares matrix: full	Hydrogen site location: difference Fourier map
*R*[*F*^2^ > 2σ(*F*^2^)] = 0.041	H-atom parameters constrained
*wR*(*F*^2^) = 0.096	*w* = 1/[σ^2^(*F*_o_^2^) + (0.0426*P*)^2^ + 0.2167*P*] where *P* = (*F*_o_^2^ + 2*F*_c_^2^)/3
*S* = 1.03	(Δ/σ)_max_ < 0.001
4503 reflections	Δρ_max_ = 0.33 e Å^−^^3^
227 parameters	Δρ_min_ = −0.25 e Å^−^^3^
1 restraint	Absolute structure: Flack (1983), 2131 Friedel pairs
0 constraints	Flack parameter: −0.07 (7)
Primary atom site location: structure-invariant direct methods	

### Fractional atomic coordinates and isotropic or equivalent isotropic displacement parameters (Å^2^)

**Table:**

	*x*	*y*	*z*	*U*_iso_*/*U*_eq_	
S1	0.56575 (3)	0.02701 (3)	0.35040 (5)	0.02691 (11)	
F1	0.77633 (15)	0.11534 (12)	−0.03264 (18)	0.0792 (6)	
O1	0.46206 (10)	0.30803 (10)	0.29361 (13)	0.0277 (3)	
O2	0.56405 (11)	−0.00184 (12)	0.48474 (14)	0.0390 (4)	
C1	0.49571 (12)	0.14257 (13)	0.33717 (18)	0.0234 (3)	
C2	0.40393 (13)	0.17197 (14)	0.40656 (17)	0.0231 (3)	
C3	0.33673 (13)	0.12486 (14)	0.49109 (16)	0.0254 (4)	
H3	0.3475	0.0555	0.5143	0.031*	
C4	0.25446 (14)	0.17975 (15)	0.54083 (17)	0.0257 (4)	
C5	0.24014 (14)	0.28249 (15)	0.5060 (2)	0.0296 (4)	
H5	0.1833	0.3196	0.5408	0.036*	
C6	0.30589 (14)	0.33145 (15)	0.42275 (19)	0.0287 (4)	
H6	0.2957	0.4009	0.3997	0.034*	
C7	0.38672 (13)	0.27399 (14)	0.37531 (16)	0.0260 (4)	
C8	0.52756 (14)	0.22666 (14)	0.27291 (16)	0.0256 (4)	
C9	0.17818 (14)	0.12816 (16)	0.62864 (17)	0.0287 (4)	
H9	0.2064	0.0585	0.6469	0.034*	
C10	0.16664 (19)	0.1834 (2)	0.7521 (2)	0.0443 (5)	
H10A	0.1439	0.2547	0.7367	0.053*	
H10B	0.2354	0.1858	0.7943	0.053*	
C11	0.08727 (19)	0.1306 (2)	0.8365 (2)	0.0554 (7)	
H11A	0.1153	0.0634	0.8625	0.067*	
H11B	0.0767	0.1725	0.9121	0.067*	
C12	−0.01698 (19)	0.1153 (2)	0.7722 (3)	0.0573 (8)	
H12A	−0.0642	0.0761	0.8273	0.069*	
H12B	−0.0495	0.1827	0.7564	0.069*	
C13	−0.00474 (18)	0.0591 (2)	0.6512 (3)	0.0494 (6)	
H13A	−0.0736	0.0540	0.6095	0.059*	
H13B	0.0206	−0.0112	0.6676	0.059*	
C14	0.07219 (16)	0.1139 (2)	0.5661 (2)	0.0405 (5)	
H14A	0.0434	0.1816	0.5431	0.049*	
H14B	0.0813	0.0737	0.4890	0.049*	
C15	0.61838 (13)	0.24653 (16)	0.19341 (17)	0.0269 (4)	
C16	0.65392 (17)	0.17134 (18)	0.1135 (2)	0.0389 (5)	
H16	0.6172	0.1086	0.1051	0.047*	
C17	0.74380 (18)	0.18950 (19)	0.0462 (2)	0.0457 (6)	
C18	0.79812 (17)	0.27964 (19)	0.0536 (2)	0.0421 (5)	
H18	0.8604	0.2896	0.0069	0.051*	
C19	0.76030 (16)	0.35559 (19)	0.1303 (2)	0.0376 (5)	
H19	0.7957	0.4194	0.1349	0.045*	
C20	0.67083 (14)	0.33938 (16)	0.20081 (19)	0.0306 (4)	
H20	0.6455	0.3917	0.2541	0.037*	
C21	0.47422 (18)	−0.05631 (16)	0.2771 (2)	0.0378 (5)	
H21A	0.5009	−0.1266	0.2794	0.057*	
H21B	0.4644	−0.0352	0.1905	0.057*	
H21C	0.4071	−0.0530	0.3209	0.057*	

### Atomic displacement parameters (Å^2^)

**Table:**

	*U*^11^	*U*^22^	*U*^33^	*U*^12^	*U*^13^	*U*^23^
S1	0.02477 (19)	0.0252 (2)	0.0307 (2)	0.00363 (16)	0.00124 (19)	0.0016 (2)
F1	0.0930 (12)	0.0564 (10)	0.0883 (13)	0.0007 (9)	0.0641 (10)	−0.0086 (9)
O1	0.0257 (6)	0.0243 (7)	0.0332 (7)	−0.0006 (5)	0.0024 (5)	0.0051 (6)
O2	0.0457 (9)	0.0377 (8)	0.0335 (8)	0.0094 (7)	−0.0076 (7)	0.0065 (7)
C1	0.0219 (7)	0.0237 (8)	0.0246 (9)	−0.0008 (6)	0.0011 (7)	0.0004 (8)
C2	0.0207 (7)	0.0243 (9)	0.0243 (8)	0.0011 (7)	−0.0022 (6)	0.0010 (7)
C3	0.0255 (8)	0.0236 (9)	0.0272 (9)	0.0014 (7)	0.0001 (7)	0.0023 (7)
C4	0.0246 (9)	0.0267 (10)	0.0256 (9)	0.0020 (7)	0.0008 (7)	0.0007 (7)
C5	0.0248 (9)	0.0291 (10)	0.0350 (10)	0.0042 (7)	0.0020 (7)	−0.0021 (8)
C6	0.0279 (9)	0.0232 (9)	0.0352 (10)	0.0026 (7)	−0.0013 (8)	0.0013 (8)
C7	0.0219 (8)	0.0262 (9)	0.0301 (10)	−0.0030 (7)	−0.0022 (7)	0.0023 (7)
C8	0.0244 (8)	0.0264 (10)	0.0260 (9)	0.0005 (7)	−0.0019 (7)	0.0011 (7)
C9	0.0284 (9)	0.0306 (10)	0.0271 (9)	0.0055 (7)	0.0042 (7)	0.0033 (8)
C10	0.0440 (12)	0.0575 (15)	0.0315 (11)	−0.0056 (11)	0.0084 (9)	−0.0057 (10)
C11	0.0643 (15)	0.0647 (17)	0.0373 (13)	−0.0022 (12)	0.0242 (12)	−0.0026 (13)
C12	0.0434 (13)	0.0520 (15)	0.077 (2)	0.0069 (12)	0.0309 (13)	0.0186 (14)
C13	0.0353 (11)	0.0559 (16)	0.0571 (15)	−0.0109 (11)	0.0033 (10)	0.0208 (12)
C14	0.0383 (12)	0.0457 (14)	0.0376 (11)	−0.0115 (10)	−0.0014 (9)	0.0124 (10)
C15	0.0250 (8)	0.0297 (10)	0.0261 (9)	0.0002 (7)	0.0008 (7)	0.0066 (7)
C16	0.0445 (12)	0.0335 (12)	0.0387 (12)	−0.0035 (9)	0.0140 (9)	0.0040 (9)
C17	0.0474 (13)	0.0440 (14)	0.0457 (13)	0.0073 (11)	0.0228 (11)	0.0087 (11)
C18	0.0297 (10)	0.0564 (15)	0.0402 (12)	0.0000 (10)	0.0101 (9)	0.0163 (11)
C19	0.0295 (10)	0.0448 (13)	0.0386 (11)	−0.0125 (9)	−0.0027 (9)	0.0095 (10)
C20	0.0289 (9)	0.0315 (11)	0.0312 (10)	−0.0024 (8)	−0.0044 (8)	0.0049 (8)
C21	0.0447 (12)	0.0281 (10)	0.0406 (12)	−0.0025 (9)	−0.0070 (9)	−0.0039 (9)

### Geometric parameters (Å, º)

**Table:**

S1—O2	1.4954 (15)	C11—C12	1.515 (4)
S1—C1	1.7620 (17)	C11—H11A	0.9900
S1—C21	1.783 (2)	C11—H11B	0.9900
F1—C17	1.355 (3)	C12—C13	1.505 (4)
O1—C8	1.372 (2)	C12—H12A	0.9900
O1—C7	1.378 (2)	C12—H12B	0.9900
C1—C8	1.361 (3)	C13—C14	1.523 (3)
C1—C2	1.443 (2)	C13—H13A	0.9900
C2—C7	1.393 (3)	C13—H13B	0.9900
C2—C3	1.395 (2)	C14—H14A	0.9900
C3—C4	1.381 (2)	C14—H14B	0.9900
C3—H3	0.9500	C15—C16	1.383 (3)
C4—C5	1.407 (3)	C15—C20	1.389 (3)
C4—C9	1.516 (3)	C16—C17	1.378 (3)
C5—C6	1.385 (3)	C16—H16	0.9500
C5—H5	0.9500	C17—C18	1.370 (3)
C6—C7	1.376 (3)	C18—C19	1.379 (3)
C6—H6	0.9500	C18—H18	0.9500
C8—C15	1.465 (2)	C19—C20	1.389 (3)
C9—C10	1.520 (3)	C19—H19	0.9500
C9—C14	1.524 (3)	C20—H20	0.9500
C9—H9	1.0000	C21—H21A	0.9800
C10—C11	1.527 (3)	C21—H21B	0.9800
C10—H10A	0.9900	C21—H21C	0.9800
C10—H10B	0.9900		
			
O2—S1—C1	106.69 (9)	C10—C11—H11B	109.3
O2—S1—C21	105.37 (10)	H11A—C11—H11B	107.9
C1—S1—C21	98.93 (9)	C13—C12—C11	111.69 (19)
C8—O1—C7	106.23 (14)	C13—C12—H12A	109.3
C8—C1—C2	106.92 (15)	C11—C12—H12A	109.3
C8—C1—S1	125.59 (13)	C13—C12—H12B	109.3
C2—C1—S1	126.83 (13)	C11—C12—H12B	109.3
C7—C2—C3	118.91 (16)	H12A—C12—H12B	107.9
C7—C2—C1	104.97 (15)	C12—C13—C14	111.0 (2)
C3—C2—C1	136.11 (17)	C12—C13—H13A	109.4
C4—C3—C2	119.48 (17)	C14—C13—H13A	109.4
C4—C3—H3	120.3	C12—C13—H13B	109.4
C2—C3—H3	120.3	C14—C13—H13B	109.4
C3—C4—C5	119.46 (17)	H13A—C13—H13B	108.0
C3—C4—C9	120.00 (17)	C13—C14—C9	111.42 (18)
C5—C4—C9	120.51 (16)	C13—C14—H14A	109.3
C6—C5—C4	122.35 (17)	C9—C14—H14A	109.3
C6—C5—H5	118.8	C13—C14—H14B	109.3
C4—C5—H5	118.8	C9—C14—H14B	109.3
C7—C6—C5	116.31 (17)	H14A—C14—H14B	108.0
C7—C6—H6	121.8	C16—C15—C20	119.90 (18)
C5—C6—H6	121.8	C16—C15—C8	119.84 (18)
C6—C7—O1	125.82 (17)	C20—C15—C8	120.22 (18)
C6—C7—C2	123.48 (17)	C17—C16—C15	118.6 (2)
O1—C7—C2	110.69 (15)	C17—C16—H16	120.7
C1—C8—O1	111.19 (15)	C15—C16—H16	120.7
C1—C8—C15	132.60 (17)	F1—C17—C18	119.79 (19)
O1—C8—C15	116.14 (16)	F1—C17—C16	117.5 (2)
C4—C9—C10	113.34 (18)	C18—C17—C16	122.7 (2)
C4—C9—C14	110.48 (16)	C17—C18—C19	118.5 (2)
C10—C9—C14	111.02 (18)	C17—C18—H18	120.8
C4—C9—H9	107.2	C19—C18—H18	120.8
C10—C9—H9	107.2	C18—C19—C20	120.4 (2)
C14—C9—H9	107.2	C18—C19—H19	119.8
C9—C10—C11	111.7 (2)	C20—C19—H19	119.8
C9—C10—H10A	109.3	C19—C20—C15	119.9 (2)
C11—C10—H10A	109.3	C19—C20—H20	120.0
C9—C10—H10B	109.3	C15—C20—H20	120.0
C11—C10—H10B	109.3	S1—C21—H21A	109.5
H10A—C10—H10B	107.9	S1—C21—H21B	109.5
C12—C11—C10	111.8 (2)	H21A—C21—H21B	109.5
C12—C11—H11A	109.3	S1—C21—H21C	109.5
C10—C11—H11A	109.3	H21A—C21—H21C	109.5
C12—C11—H11B	109.3	H21B—C21—H21C	109.5
			
O2—S1—C1—C8	133.15 (16)	C7—O1—C8—C15	−176.61 (15)
C21—S1—C1—C8	−117.75 (17)	C3—C4—C9—C10	125.5 (2)
O2—S1—C1—C2	−36.29 (18)	C5—C4—C9—C10	−56.7 (2)
C21—S1—C1—C2	72.81 (18)	C3—C4—C9—C14	−109.2 (2)
C8—C1—C2—C7	1.11 (19)	C5—C4—C9—C14	68.6 (2)
S1—C1—C2—C7	172.15 (13)	C4—C9—C10—C11	178.61 (19)
C8—C1—C2—C3	−177.8 (2)	C14—C9—C10—C11	53.6 (3)
S1—C1—C2—C3	−6.8 (3)	C9—C10—C11—C12	−53.3 (3)
C7—C2—C3—C4	0.6 (3)	C10—C11—C12—C13	54.5 (3)
C1—C2—C3—C4	179.4 (2)	C11—C12—C13—C14	−55.9 (3)
C2—C3—C4—C5	−0.4 (3)	C12—C13—C14—C9	56.4 (3)
C2—C3—C4—C9	177.44 (16)	C4—C9—C14—C13	178.2 (2)
C3—C4—C5—C6	0.0 (3)	C10—C9—C14—C13	−55.2 (3)
C9—C4—C5—C6	−177.75 (18)	C1—C8—C15—C16	39.0 (3)
C4—C5—C6—C7	0.0 (3)	O1—C8—C15—C16	−144.42 (19)
C5—C6—C7—O1	−178.59 (17)	C1—C8—C15—C20	−139.0 (2)
C5—C6—C7—C2	0.2 (3)	O1—C8—C15—C20	37.6 (2)
C8—O1—C7—C6	178.97 (17)	C20—C15—C16—C17	2.5 (3)
C8—O1—C7—C2	0.04 (19)	C8—C15—C16—C17	−175.5 (2)
C3—C2—C7—C6	−0.5 (3)	C15—C16—C17—F1	−179.1 (2)
C1—C2—C7—C6	−179.67 (17)	C15—C16—C17—C18	−1.3 (4)
C3—C2—C7—O1	178.43 (15)	F1—C17—C18—C19	176.9 (2)
C1—C2—C7—O1	−0.7 (2)	C16—C17—C18—C19	−0.9 (4)
C2—C1—C8—O1	−1.1 (2)	C17—C18—C19—C20	1.8 (3)
S1—C1—C8—O1	−172.33 (13)	C18—C19—C20—C15	−0.6 (3)
C2—C1—C8—C15	175.59 (19)	C16—C15—C20—C19	−1.6 (3)
S1—C1—C8—C15	4.4 (3)	C8—C15—C20—C19	176.39 (18)
C7—O1—C8—C1	0.71 (19)		

### Hydrogen-bond geometry (Å, º)

**Table:**

*D*—H···*A*	*D*—H	H···*A*	*D*···*A*	*D*—H···*A*
C21—H21*B*···O2^i^	0.98	2.30	3.276 (3)	176

Symmetry code: (i) −*x*+1, −*y*, *z*−1/2.
